# Non-Invasive Brain Stimulation for the Modulation of Aggressive Behavior—A Systematic Review of Randomized Sham-Controlled Studies

**DOI:** 10.3390/life13051220

**Published:** 2023-05-20

**Authors:** Antony Casula, Bianca M. Milazzo, Gabriella Martino, Alessandro Sergi, Chiara Lucifora, Francesco Tomaiuolo, Angelo Quartarone, Michael A. Nitsche, Carmelo M. Vicario

**Affiliations:** 1 Dipartimento di Scienze Cognitive, Psicologiche, Pedagogiche e Degli Studi Culturali, Università di Messina, 98121 Messina, Italy; antonycasula@gmail.com (A.C.);; 2 Dipartimento di Medicina e Clinica Sperimentale, Università degli Studi di Messina, A.O.U. “G. Martino”, Via Consolare Valeria, 98125 Messina, Italy; gabriella.martino@unime.it (G.M.); francesco.tomaiuolo@unime.it (F.T.); 3 Dipartimento di Scienze Matematiche e Informatiche, Scienze Fisiche e Scienze della Terra, Università degli Studi di Messina, Viale F. Stagno d’Alcontres 31, 98166 Messina, Italy; alessandro.sergi@unime.it; 4 Dipartimento di Filosofia e Comunicazione, Università di Bologna, 40131 Bologna, Italy; chiara.lucifora@unibo.it; 5 IRCCS Centro Neurolesi Bonino Pulejo, 98121 Messina, Italy; angelo.quartarone@unime.it; 6 Department of Psychology and Neurosciences, Leibniz Research Centre for Working Environment and Human Factors, 44139 Dortmund, Germany; nitsche@ifado.de; 7 University Clinic of Psychiatry and Psychotherapy and University Clinic of Child and Adolescent Psychiatry and Psychotherapy, Protestant Hospital of Bethel Foundation, University Hospital OWL, Bielefeld University, 33615 Bielefeld, Germany

**Keywords:** non-invasive brain stimulation, aggression, tDCS, rTMS, cTBS

## Abstract

Intro: Aggressive behavior represents a significant public health issue, with relevant social, political, and security implications. Non-invasive brain stimulation (NIBS) techniques may modulate aggressive behavior through stimulation of the prefrontal cortex. Aims: To review research on the effectiveness of NIBS to alter aggression, discuss the main findings and potential limitations, consider the specifics of the techniques and protocols employed, and discuss clinical implications. Methods: A systematic review of the literature available in the PubMed database was carried out, and 17 randomized sham-controlled studies investigating the effectiveness of NIBS techniques on aggression were included. Exclusion criteria included reviews, meta-analyses, and articles not referring to the subject of interest or not addressing cognitive and emotional modulation aims. Conclusions: The reviewed data provide promising evidence for the beneficial effects of tDCS, conventional rTMS, and cTBS on aggression in healthy adults, forensic, and clinical samples. The specific stimulation target is a key factor for the success of stimulation on aggression modulation. rTMS and cTBS showed opposite effects on aggression compared with tDCS. However, due to the heterogeneity of stimulation protocols, experimental designs, and samples, we cannot exclude other factors that may play a confounding role.

## 1. Introduction

Human aggression consists of verbally or physically abusive behaviors with the clear intent of causing harm to another individual [1]. The intentional component (relevance of the goals instead of the results of the action) is crucial for distinguishing between aggression and accidents or other harmful behaviors (e.g., a surgical procedure performed by a surgeon with the intention to help the patient).

Although aggression might have been crucial for human evolution and survival [2,3] and is considered, in specific contexts, as adaptive behavior [4,5], some of its consequences represent a major problem for public health by affecting billions of people’s lives each year and costing approximately 11% of the world’s gross domestic product [6,7].

For this reason, aggression and its behavioral consequences qualify as hot topics in media communication and political agendas in most countries, fueling the debate about the causes of and solutions for a complex and misplaced problem [8].

Aggression can be classified into two types: proactive (also known as instrumental) aggression, which is premeditated and motivated by a specific goal, and reactive (or hostile) aggression, which is impulsive, unplanned, and triggered by provocation and anger [1,9].

### 1.1. Aggression Models

The investigation of aggression, including its underlying motivations and causative factors, holds significant importance across numerous academic fields, including behavioral genetics, evolution, neuroscience, psychology, sociology, and criminology [2]. Several models were developed to account for aggression causes, triggers, and properties [1], as discussed below.

The frustration–aggression hypothesis, initially proposed by Dollard et al. in 1939 [10], posits that aggressive behavior is always linked to frustration, and conversely, the presence of frustration always elicits some form of aggression. More recently, Breuer and Elson [11] extended the theory in the light of recent findings, addressing its limitations and discussing different antecedents of frustration (such as expectations and goal relevance) and different causes of aggression (such as the instrumental value of the aggressive act, adherence to social norms, and empathy).

The cognitive neoassociation theory [12] proposes that negative emotions, such as anger and fear, are triggered by unpleasant experiences, such as frustration, loud noises, and unpleasant odors. These negative experiences lead to automatic thoughts, memory concepts, and physiological responses associated with both fight and flight tendencies, which, in turn, lead to feelings of anger or fear. According to this model, cues present during the unpleasant event become associated with the event, cognitive and emotional responses, and aggressive thoughts and behavior, and the priming or activation of a single concept will spread to related concepts and increase their activation.

The excitation transfer theory [13] suggests that the physiological arousal increases caused by an event can last for several minutes after cognitive readaptation, affecting the following events. Arousal can be misattributed to a following event if the two events are closely temporally connected. If the second event was already related to anger beforehand, this additional arousal will increase the person’s anger. If the arousal is consciously attributed to anger, the person may remain primed for aggression even after the arousal has dissipated.

Social learning theories [14,15,16] propose that people acquire aggressive behavior through direct experience or observation of others’ behavior. These theories focus on how beliefs and expectations guide social behavior, particularly in the acquisition of aggressive behavior. Key concepts such as expectations and the interpretation of the social world are crucial for understanding instrumental aggression.

Script theory [17] proposes that children learn aggressive behavior through observing violence in the mass media, which they internalize as “scripts”, which are sets of well-rehearsed concepts in memory. Scripts guide behavior by defining situations and serving as a blueprint for action. Frequently rehearsed scripts gain accessibility strength, making them readily available and more likely to be used in future situations. This theory explains the generalization of social learning processes and the automatization of complex behavioral processes.

According to social interaction theory [18], aggressive behavior can be seen as a form of social influence, where an actor uses coercion to change the target’s behavior. The choice to engage in aggressive behavior is driven by the expected rewards, costs, and outcomes of the actor’s actions. This theory suggests that aggressive acts can serve various purposes, such as obtaining something valuable, seeking retribution, or achieving desired social or self-identities.

The general aggression model [1] provides a framework that integrates different theories of aggression, proposing that knowledge structures develop from experience and play a major role in shaping perceptions, beliefs, and responses to the social and physical environment. These knowledge structures include perceptual, person, and behavioral schemas, as well as affective states and emotions. When activated, these structures trigger the experience of emotions. They also contain knowledge about emotions and may include affect as an action rule. For example, an aggressive response may be produced by a personal insult script only if anger is high and fear is low. The general aggression model focuses on the role of the individual in the context, which is referred to as an episode, in a recurring social interaction. Factors that can trigger aggression include situational (provocations, frustration, pain, discomfort, drugs, and incentives for being aggressive), cognitive (hostile thoughts, action rules), and affective (anger) factors, as well as arousal levels, personal traits, attitudes, values, and long-term goals [1].

### 1.2. Neural Substrates of Aggression

Most relevant insights about the neural substrates of aggression come from animal models. A recent review conducted by Lischinsky and Lin [19] across species proposed a network of interconnected nuclei as a core aggression circuit (CAC). The CAC includes the medial amygdala, the ventrolateral portion of the ventromedial hypothalamus, the posterior part of the bed nucleus of the stria terminalis, and the ventral part of the premammillary nucleus. According to the CAC model, external stimuli trigger medial amygdala neurons, which receive direct input from thalamic multisensorial pathways, and have a direct role in driving aggression. The medial amygdala has dense, bidirectional connections to the posterior part of the bed nucleus of the stria terminalis, which also receives direct multisensorial input from the thalamus, and is thought to drive aggressive arousal but not physical attack [20]. Both the medial amygdala and the posterior part of the bed nucleus of the stria terminalis send direct projections to the ventrolateral portion of the ventromedial hypothalamus, whose stimulation triggers physical attacks toward natural targets [21], whereas its inactivation or ablation inhibits natural inter-male and maternal aggression in mice [22]. Evidence for this role of the ventrolateral portion of the ventromedial hypothalamus in aggression was shown across several mammal species, including humans [23,24,25]. Another nucleus included in the CAC is the ventral part of the premammillary nucleus, which provides strong excitatory projections to the ventrolateral portion of the ventromedial hypothalamus. It may play a role in adjusting aggressiveness in animals according to several factors, including seasonal changes [26].

The periaqueductal gray is involved in aggression expression. It receives dense projections from the ventrolateral portion of the ventromedial hypothalamus and projects to motor neurons in the spinal cord and is connected to the jaw muscles. Moreover, the periaqueductal gray activity is related to attack behavior since pharmacologically inhibiting the periaqueductal gray in mice results in a strong approach to the opponent but a failure to bite it (an innate attack behavior) [27].

All these deep nuclei are interconnected and form a unified circuit that has its own genetic, neurochemical, and behavioral correlates [28]. The hypothesis of a unified circuit is strengthened by the fact that aggression can be activated and suppressed by acting on each of these regions [19,29,30]. An example in humans is provided by Franzini et al. [31], who reduced aggression in a group of patients with refractory aggressive behavior and intellectual disability by employing deep brain stimulation (DBS) of the posterior hypothalamus.

Cortical contributions to aggression genesis might involve focusing aggression toward specific goals, with left-frontal areas involved in anger-related approach behavior [32].

### 1.3. Aggression Control

The existence of areas and circuits for aggression enactment requires areas and circuits for their flexible control based on the goal, the context, and the need for energy saving. The first candidate for this top-down control role is the prefrontal cortex (PFC), which is located in the rostral part of the frontal lobe. The PFC can be subdivided into 6 subregions: the dorsolateral prefrontal cortex (DLPFC, which includes the lateral Brodmann areas 9, 10, and 46), the ventrolateral prefrontal cortex (VLPFC, including the lateral Brodmann areas 10, 45, 46, and 47), the orbitofrontal cortex (OFC, including Brodmann areas 11, 12, 13, 14, 25, and 47), the anterior cingulate cortex (ACC, which includes the medial Brodmann areas 24, 32, and 33), the dorsomedial prefrontal cortex (DMPFC, including the medial Brodmann areas 8, 9, and 32), and the ventromedial prefrontal cortex (VMPFC, including the medial Brodmann areas 10 and 11) [33,34,35,36].

**Figure 1:**
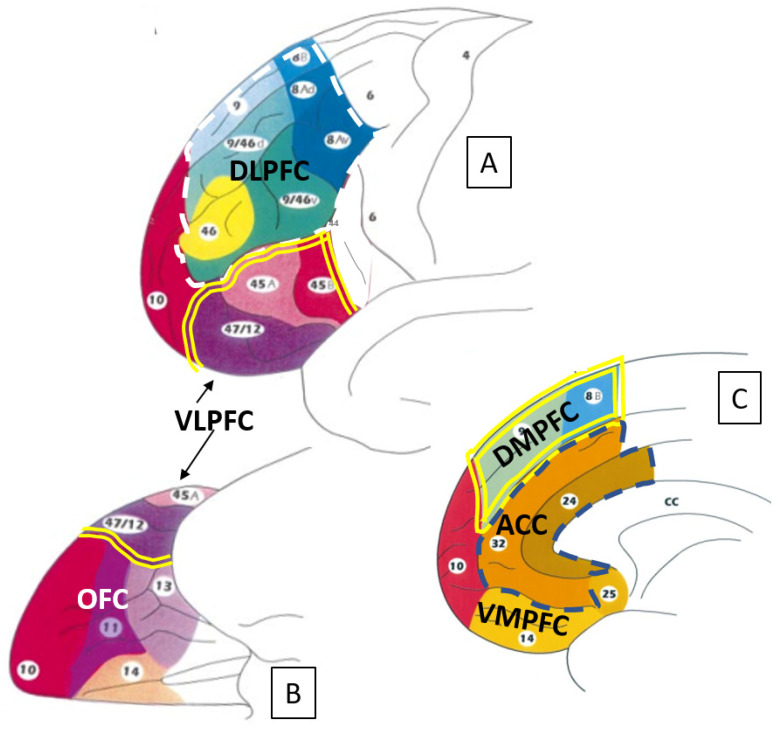
Lateral, orbital and medial surfaces of the frontal lobe in the cytoarchitectonic atlas of Petrides and Pandya [37,38]. In the present figure, the various areas in the different regions are outlined as indicated below. (**A**) Lateral aspect of the frontal lobe. The outline of the dorsolateral prefrontal cortex (DLPFC) is indicated by the white dashed lines. The outline of the ventrolateral prefrontal cortex (VLPFC) is indicated by the yellow outline. Notice that the VLPFC can be viewed also from the orbital aspect, but it is not part of the orbital frontal cortex. (**B**) Orbital aspect of the frontal lobe. The cortex on the orbital surface includes areas 14 and 11 and also the orbital extension of frontopolar area 10 and ventromedial area 14. (**C**) Medial aspect of the frontal lobe. The dorsomedial prefrontal cortex (DMPFC) includes medial areas 8 and 9. The anterior cingulate region (ACC) includes areas 24 and 32. The ventromedial prefrontal cortex (VMPFC) is area 14.

An overview of the functional subdivisions of the PFC is shown in Figure 1.

It was hypothesized that the PFC plays a major role in controlling aggressive behavior by providing inhibitory inputs to CAC regions, such as the hypothalamus and amygdala [28,39]. The key PFC regions involved in this process include the OFC, ACC, MPFC, VLPFC, and DLPFC, which have extensive mutual connections, as well as connections with various brain regions outside the PFC, such as the amygdala, the hypothalamus, the ventral tegmental area, and central autonomic structures [40,41,42,43,44].

These connections allow different regions of the PFC to be broadly involved in executive functions and top-down control of behavior (including aggression). In particular, the DLPFC is crucial for attention control, self-regulation, planning, cognitive control, working memory, time processing, and decision-making [45,46,47,48]. The VLPFC is essential for sustaining cognitive control in different domains, such as motor control, decision-making under risk, memory inhibition, and emotion regulation and control [49,50,51,52,53]. The MPFC is known as a crucial cognitive–affective integration area, where it is involved in self-related reflection, reward-based decision-making, empathy, and theory of mind (i.e., the ability to represent and understand another person’s psychological perspective by attributing mental states) [33,54]. The ACC represents a key region for flexible performance, error, and conflict monitoring [55]. Finally, the OFC is involved in stimulus–reinforcement association devaluation, cognitive mapping (generating and updating a model of the environment for prediction purposes) [56], social behavior, and morality [56,57,58,59]. Regarding this connection, in his somatic marker hypothesis, Damasio [60] described the OFC (especially its ventromedial portion) as a crucial area for linking emotionally relevant information with contextual information, as well as somatic feelings for behavioral modulation in social contexts.

The relation between aggressive behavior and prefrontal dysfunction was documented in humans by several studies [61,62]. A meta-analysis of 43 independent structural and functional imaging studies [63] found that antisocial individuals showed reduced gray or white tissue volume, hemodynamic response, and regional cerebral blood flow of the left DLPFC, right OFC, and right ACC.

Two functional magnetic resonance imaging (fMRI) studies showed the role of the VLPFC in inhibiting amygdala activity during the reappraisal of adverse images [64,65]. Further studies delivered evidence for the important role of the OFC and VMPFC in the inhibition of anger and aggressive behavior, most likely due to their role in mediating empathy with the potential victim of the aggression [66]. The role of the MPFC in anger and aggression modulation was shown in both animal and human models [67,68,69]. Optogenetic studies in mice showed that inhibition of the MPFC increases aggression, whereas its activation reduces inter-male aggression [69]. Additionally, the relationship between the MPFC and aggression is mediated by serotonin levels: aggression increases with serotonin deficiency in the MPFC of mice [70]

Some papers also reported an association between aggressive behavior and hypometabolism and reduced tissue volume of the temporal cortex [66,71]. A hypothesis about a differential role of the PFC and temporal cortex in aggression postulates that aggressive responses directed to a target are associated with frontal lobe damage, whereas intense but unfocused anger and aggression are linked with temporal lobe dysfunction [72].

The relationship between PFC functions and aggression may be explained by the fact that self-control failure is a predictor of aggression, and self-control mainly involves the DLPFC. Thus, DLPFC dysfunction might result in deficient control of anger, and aggressive drives in case of provocation [73].

Two models aimed to identify neural correlates of aggression-control failures are given below.

The motivational direction model of frontal asymmetry (MDMFA) [32,74,75] proposes that avoidance and withdrawal are associated with right-frontal activity, and anger-related approach behavior is associated with left-frontal activity. According to this model, anger and aggression problems can be conceived as a consequence of frontal activity asymmetry, particularly hyperactivation of the left PFC and/or hypoactivation of the right PFC.

The triple imbalance hypothesis (I3) [76] focalizes on the neural underpinnings of reactive aggression, postulating that hormonal dysbalance (mainly high levels of testosterone and low levels of cortisol) leads to a functional dysbalance of cortical–subcortical and hemispheric relations, compromising self-control. This hormonal imbalance concept proposes hyperactivation of the hypothalamic–pituitary–gonadal axis (HPG) or/and hypoactivation of the hypothalamic–pituitary–adrenal axis (HPA), resulting in a high testosterone–cortisol ratio. This anomalous hormonal and cerebral activity pattern is supposed to produce a dominance of reward-seeking over punishment–withdrawal processes, favoring aggressive behavior and hindering the individual from being guided by subcortical input (i.e., CAC) in socially and morally appropriate ways. This theory is tightly connected with the triple balance hypothesis [77], which postulates endocrine–autonomic homeostasis as the foundation for sustaining subcortical, cortical, and subcortico–cortical balance and approach–withdrawal behavior. Finally, this model incorporates the Harmon–Jones motivational direction model of frontal asymmetry, hypothesizing a link between hormonal dysbalance and prefrontal hemispheric asymmetry as a cause for dysfunctional control of reactive aggression [76].

It was argued that different types of aggression (i.e., proactive and reactive) have distinct neural correlates. Specifically, proactive aggression, which is characterized by motivation directed to specific goals, involves areas specialized in planning and goal setting, such as the DLPFC and the ACC, while reactive aggression, which is characterized by impulsiveness and lack of planning, primarily involves areas relevant for emotion processing and threat assessment, such as the OFC and the amygdala [78,79,80].

### 1.4. NIBS

The acronym NIBS (i.e., non-invasive brain stimulation) includes several techniques capable of modulating cortical activity in a non-invasive way. In the current article, we focused on transcranial direct current stimulation (tDCS) and repetitive transcranial magnetic stimulation (rTMS), which are the two main techniques employed for modulating aggressive behavior in humans.

tDCS is a neuromodulatory brain stimulation technique involving two or more electrodes, with at least one placed on the scalp, connected to a direct current generator that produces a weak (usually 1–2 mA) electrical direct current via a cathode (i.e., negative pole) and an anode (i.e., positive pole) [81]. The application of respective direct currents influences the excitability of neurons by modulating their resting membrane potential [81,82,83]. With conventional protocols, at the macroscale level, anodal tDCS increases neuronal excitability, resulting in increased spontaneous neural network activity. In contrast, cathodal tDCS decreases neuronal excitability and spontaneous firing rates [84]. Sufficiently long stimulation for a few minutes induces neuroplastic after-effects of stimulation, which can last for hours, and with repeated stimulation, can last even longer [81,84,85,86]. The long-term effects of tDCS depend on the glutamatergic system, are calcium-dependent, are gated by a reduction of GABA activity, and share mechanisms of long-term potentiation (LTP) and long-term depression (LTD) [86,87,88,89,90].

TMS is based on the induction of an electric field in the brain via a strong magnetic pulse applied by a copper coil positioned over the target region [91,92]. Single pulses of sufficient intensity cause the induction of action potentials using suprathreshold activation that, if applied over the motor cortex, produces immediate twitches in a target muscle, evoking motor-evoked potentials (MEP) monitored via electromyography. This measure is relevant to study the contribution of the motor system in different psychological processes, such as associative learning, and the processing of negative emotions, such as disgust and regret [93,94,95,96], and can be used for producing transient and reversible “virtual lesions” by disrupting cortical activity in specific areas involved in task-relevant functions when applied with appropriate timing [97,98]. Moreover, repetitive TMS (rTMS) protocols can induce long-lasting after-effects depending on the stimulation frequency, intensity, coil shape, and duration of the stimulation period [99,100,101,102]. Stimulation of the targeted cortex with high-frequency rTMS (10–20 Hz) can lead to an increase in neuronal activity and excitability [100,103]. On the other hand, low-frequency (1 Hz) rTMS results in a decrease in neuronal activity and excitability [100,104]. The neurochemical mechanisms involved in the long-lasting effects of rTMS have not yet been completely unveiled. However, they seem to be similar to those observed in vitro after the electrical induction of LTP and LTD, as they are mediated by GABAergic (γ-aminobutyric acid) and NMDA (N-methyl-d-aspartate) receptor-dependent glutamatergic transmission [105,106].

A newly developed rTMS protocol, namely, theta-burst stimulation (TBS), is based on the naturally occurring theta rhythm of the hippocampus (5 Hz) [105,107], and on an electrical stimulation protocol that is widely used in animal experiments [108]. It consists of triple-pulse 50 Hz bursts delivered at a rate of 5 Hz (i.e., 200 ms between each burst). Different TBS protocols are known for producing a long-lasting enhancement or reduction of cortical excitability by varying the stimulus sequence (respectively, intermittent or iTBS, and continuous or cTBS) [100,107,109,110]. It was hypothesized that both intervention protocols involve both excitatory and inhibitory pathways, but differ in the final balance and produce opposite effects [109,111]. TBS effects are GABA-, NMDA receptor-, and calcium-dependent, corroborating the hypothesis of LTP- and LTD-like mechanisms for the genesis of its effects [109,112].

The interest in NIBS techniques has recently grown fast, with an increase in the number of publications suggesting that NIBS is a promising approach to ameliorating a wide range of mental disorders in both adults [113,114,115,116,117,118,119,120] and pediatric populations [121,122,123].

### 1.5. Effects of NIBS Targeting PFC on Behavior

Numerous tDCS, rTMS, and TBS protocols were employed for modulating behavior associated with PFC functions in humans, primarily targeting the DLPFC, VLPFC, and MPFC.

Studies involving healthy subjects provide evidence for the effectiveness of tDCS in enhancing higher cognitive functions, including sustained attention [124], working memory [125,126], behavioral control (including control over risk-taking and overeating) [127,128], affective control [129,130,131,132,133], problem-solving [134,135], inhibition [136,137], and social cognition [138].

Additionally, experiments in patients with neurological and psychiatric conditions have demonstrated a positive impact of tDCS, resulting in significant symptom reductions in several diseases, including aphasia [139,140], depression [141,142], schizophrenia [143,144], bipolar disorder [145], Parkinson’s disease (executive deficits) [146], and alcohol addiction [147].

Similarly, conventional rTMS studies demonstrated its efficacy in both healthy subjects and patients for modulating executive functions and behavioral and affective control [125,148,149,150,151,152], as well as reducing symptoms in patients with neurological and psychiatric conditions [153,154,155,156,157].

In contrast, TBS is a relatively new rTMS protocol and evidence is still accumulating [109]. Nonetheless, encouraging results show the significant effects of TBS on cognitive functions in healthy participants and symptom severity in patients [158,159,160]. Particularly, iTBS was found to mainly enhance neuronal excitability [160,161], while cTBS was found to impair it [161,162].

Despite these promising findings, some papers reported null effects of tDCS [163,164,165,166,167] and rTMS [168,169,170], and TBS research is still in its early stage, showing the need for further research and systematic reviews of the literature to consolidate results and address inconsistent findings.

### 1.6. Aggression Measures

In the literature, several tests and self-report questionnaires are available to assess aggressive behavior or the intent to commit harmful acts, including the Taylor Aggression Paradigm (TAP) [171,172], the Buss–Perry Aggression Questionnaire (BP-AQ) [173], the Reactive–Proactive Aggression Questionnaire (RPQ) [174], and the Voodoo Doll Task (VDT) [175]. A brief description of these measures is shown in Box 1.

Box 1 **Main tasks and questionnaires employed for measuring aggression.**

  **Taylor aggression paradigm (TAP).** A computer-based task in which participants have to compete against an opponent (which is fictitious) in a competitive reaction-time task. The winner of each trial has to choose a punishment for the loser (originally an electric shock, nowadays a noise blast via headphones). If they lose the competition, participants can see the level of the blast that their opponent selected for them, allowing the experimenters to manipulate the level of provocation experienced by participants. Aggression levels are measured as the level of the noise blasts the participant selects for the opponent [171,172].

  **Buss-Perry aggression questionnaire (BP-AQ).** A 29-item, self-report questionnaire composed of four subscales: Physical Aggression, Verbal Aggression, Anger, and Hostility. The first two subscales involve hurting or harming others (e.g., “If somebody hits me, I hit back”, “I often find myself disagreeing with people”), representing instrumental or motor components of behavior. The third subscale involves physiological arousal and preparation for aggression (e.g., “I have trouble controlling my temper”), representing the emotional or affective component of behavior. The fourth subcale consists of feelings of ill will and injustice (e.g., “I am suspicious of overly friendly strangers”), representing the cognitive component of behavior [173].

  **Reactive-proactive aggression questionnaire (RPQ).** A 23-item, self-report questionnaire (participants have to rate the frequency of specific events) composed of two subscales: Reactive Aggression and Proactive Aggression. The first is described as a response to a perceived provocation or threat, reflexive and impulsive (e.g., “Gotten angry when frustrated”). The second refers to a deliberate behavior directed towards achieving a specific goal or gaining social dominance over others (e.g., “Hurt others to win a game”) [174].

  **Voodoo doll task (VDT).** A task in which participants are exposed to a computer-generated image of a person they know, such as a friend or partner, represented by a doll. They are asked to express any negative feelings they have towards that person by inserting a certain number of pins into the doll. The more pins they insert, the greater the level of aggression they are expressing. The task is presented without any reference to “voodoo” [175].

### 1.7. AIMS

This systematic review aimed to evaluate the neuromodulatory effectiveness of non-invasive brain stimulation (NIBS) techniques for the modulation of aggression and to provide a comprehensive account for improving intervention protocols.

We examined the effectiveness of NIBS interventions for both unprovoked (i.e., proactive) and provoked (i.e., reactive) aggression elicited by experimental conditions, such as social exclusion, insult, or exposure to violent video games.

The final section addresses the limitations and respective implications for future improvements in developing effective treatment protocols.

## 2. Methods

### 2.1. Research Strategy

This systematic review adhered to the Preferred Reporting Items for Systematic Reviews and Meta-Analyses (PRISMA) guidelines, which provide a systematic checklist for helping systematic reviewers to transparently report why the review was done, what the authors did, and what they found [176,177].

Suitable publications were identified in February 2023 based on the PubMed database with no time restrictions. We used different keywords (tDCS, rTMS, theta-burst stimulation, non-invasive brain stimulation, aggressive behavior, aggression, frontal cortex, parietal cortex, temporal cortex, occipital cortex) and their combinations, identifying a total of 207 articles. Keyword combinations are shown in supplemental materials, along with the entire process description and references for all 207 examined items.

**Figure 2:**
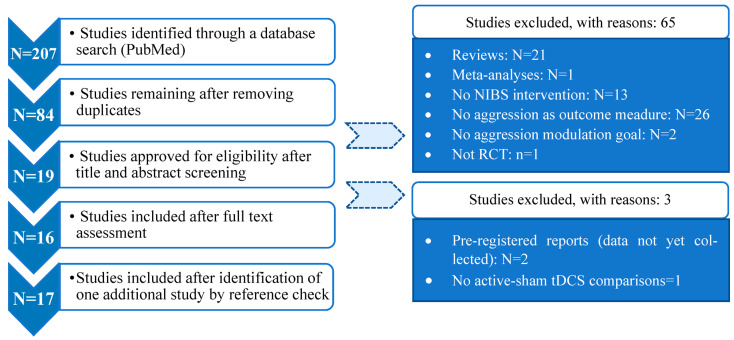
Flow chart for eligible study selection. Seventeen articles were selected as suitable based on the eligibility criteria. Studies not related to the purpose of the review were excluded. One article was identified using the citation list of already included articles.

The results of the selection process are shown in Figure 2. The initial 207 articles were reduced after the removal of duplicates (PMID comparison). A further study, which was identified by checking the literature lists of already included studies was added. The remaining 84 studies were screened based on their title and a full-text assessment, resulting in the selection of 17 articles.

### 2.2. Eligibility Criteria

The following inclusion and exclusion criteria were used for study selection to ensure a high level of methodological accuracy.

Inclusion criteria:

- Original quantitative research investigating the effectiveness of NIBS on human aggression;
- Publication in English-language peer-reviewed journals;
- Behavioral measures of aggression as an outcome;
- Comparison between active stimulation and sham control conditions;
- At least single-blinded.

Exclusion criteria:

(a) Reviews and meta-analyses;
(b) Articles not employing NIBS;
(c) Articles not conducted in humans, not referring to the subject of interest (aggression), or not addressing behavioral modulation aims.

Details of the 17 final articles included in the review are shown in the Supplemental material.

## 3. Results

The final database included 17 published studies that evaluated the effects of NIBS techniques on aggressive behavior. The cortical targets were, in most studies, the VLPFC and DLPFC. Three studies examined the role of the VMPFC.

### 3.1. Descriptive Overview by Stimulated Area

#### 3.1.1. VLPFC

The first neuromodulation study conducted by Riva et al. [178] aimed to investigate the modulatory role of a single session of tDCS over the rVLPFC on the association between social exclusion and aggression in 80 healthy university students. The participants were randomly divided into two groups, with one receiving unilateral anodal tDCS over the rVLPFC with the cathode positioned over the contralateral supraorbital region and the other receiving sham stimulation. During the last five minutes of stimulation, participants performed the Cyberball task [179], which is a virtual online ball-tossing game in which virtual players can either include or exclude participants by throwing the ball toward them or ignoring them. After stimulation (and conduction of the Cyberball task), participants performed the hot sauce paradigm [180], in which they were asked to administer a certain amount of hot sauce to the game partner after being informed that he does not like spicy food. The results showed that socially excluded participants administered more hot sauce to the game partner in comparison to non-socially-excluded participants and this effect was reduced by anodal but not sham tDCS, suggesting that the relationship between social exclusion and aggression was reduced by the intervention.

A subsequent study conducted by the same research group [181] aimed to investigate whether a single session of tDCS over the rVLPFC can reduce violent-video-game-induced aggression in 79 healthy university students. The study employed the same stimulation protocol and design as the previous one. During stimulation, participants were allowed to engage in various immoral activities while playing the video game Grand Theft Auto (GTA) or two non-violent video games (independent groups with random assignments). Afterward, they performed the TAP. The results showed that players who received anodal tDCS over the rVLPFC judged the game GTA to be more immoral than players who received the sham stimulation. In addition, anodal tDCS reduced unprovoked-proactive (but not provoked-reactive) aggression levels in violent video game players.

A further experiment [182] aimed to investigate the effects of single-session tDCS over the rVLPFC on aggression and involved 32 healthy volunteers. Participants were randomly divided into two groups, with one receiving anodal tDCS over the rVLPFC with the cathode placed over the occipital cortex and the other one receiving sham stimulation. After stimulation, participants performed the TAP. The results showed a significant difference in both proactive and reactive aggression scores of the TAP between active and sham stimulation. Participants exposed to the active tDCS were less aggressive than those exposed to the sham tDCS.

In a study conducted by Gallucci et al. [183] with 90 healthy volunteers, the aim was to investigate the effects of single-session tDCS over the VLPFC on frustration-induced aggression. The participants completed online measures of anger and aggression (Trait Anger Scale, BP-AQ) prior to the neurostimulation session. Then, they were randomly assigned to one of three conditions: (a) anodal tDCS over the rVLPFC, (b) anodal tDCS over the lVLPFC, or (c) sham tDCS. In each condition, the cathode was positioned over the contralateral supraorbital area. During the last five minutes of stimulation, participants were exposed to a frustrating number sequence task (unsolvable numerical sequences based on tests of general intelligence that were chosen by a fictitious partner), and later given a chance to behave aggressively against the same partner by performing the competitive reaction time task (CRTT), which is a variant of the TAP [184]. The results showed significantly higher aggression levels in the group exposed to anodal stimulation over the lVLPFC than after sham stimulation, whereas the baseline aggression scores did not differ between groups. In contrast, the group exposed to stimulation over the rVLPFC showed no statistically significant difference from the group exposed to sham stimulation. In addition, a significant interaction between gender and tDCS effects was found for frustration-induced aggression: women acted less aggressively than men in the sham condition but not in the active tDCS condition, regardless of the laterality of stimulation.

Dambacher et al. [185] explicitly aimed to test the MDMFA [32] by applying bicephalic bihemispheric anodal/cathodal tDCS over the VLPFC bilaterally in a single session approach to modulate aggression in 64 healthy volunteers. Participants were randomly divided into three groups: right-frontal dominance (the anode placed over the right and the cathode over the left VLPFC), left-frontal dominance (the cathode placed over the right and the anode over the left VLPFC), and sham stimulation; afterward, they performed the TAP. None of the stimulation protocols modulated aggression levels.

Smits et al. [186] provided a rehabilitation protocol by employing five sessions of tDCS over the rVLPFC in a clinical male military veterans sample (96 participants with PTSD, anxiety, or impulsive aggression). The patients were randomly divided into two groups, with one receiving anodal tDCS over the rVLPFC with the cathode over the contralateral supraorbital region and the other receiving sham stimulation. Self-reported aggression was measured only in the impulsive aggression subgroup (45 patients) using the State-Trait Anger Expression Inventory-2 (STAXI-2) [187] before and after the last stimulation session. Five sessions of anodal tDCS over the rVLPFC provided no evidence for an effect on impulsive aggression symptoms. No studies with TMS or TBS over the VLPFC are available.

#### 3.1.2. DLPFC

Several studies tested the hypothesis of a causal involvement of the DLPFC in aggression by employing neuromodulation approaches.

In the first study conducted by Hortensius et al. [188], the aim was to test the therapeutic effect of a single session of bipolar bihemispheric tDCS over the DLPFC on provoked aggression in 80 healthy volunteers. Participants were asked to write an essay about a controversial topic, which was then evaluated on a 1 to 9 scale by a fictitious partner. After writing the essay, participants were randomly divided into three groups: the first group received bipolar bihemispheric tDCS to the DLPFC (anodal lDLPFC and cathodal rDLPFC stimulation), the second group received the same stimulation with opposite polarities, and the third one received sham stimulation. Participants were then exposed to negative evaluations of the essay and insults from their partner (e.g., “I can’t believe an educated person would think like this”) and completed the TAP and anger measures. The results showed increased aggression after anodal tDCS over the lDLPFC and cathodal tDCS over the rDLPFC (but not the opposite montage or sham) in participants who scored higher on insult-related anger compared with participants who scored lower on insult-related anger.

Another study [189] investigated the modulatory effect of anodal tDCS over the rDLPFC on aggression in 32 healthy volunteers. The researchers randomly divided the participants into two groups, with one receiving anodal tDCS over the rDLPFC with the cathode positioned over the left supraorbital area and the other receiving sham stimulation. Afterward, participants performed the TAP. The results showed a statistically significant difference in proactive aggression between the group receiving active tDCS and that receiving sham stimulation in male but not female participants. Specifically, males exposed to the active tDCS exhibited a lower aggression level than males exposed to the sham tDCS.

More recently, Weidler et al. [190] aimed to replicate the study of Dambacher et al. [189] in a sample composed of 51 male alcohol abusers and healthy smokers and non-smokers. Participants were randomly assigned to one out of two groups: the first group received anodal stimulation over the rDLPFC with the cathode over the left supraorbital area, while the second one received sham stimulation. Before and after the stimulation, participants performed an adapted version of the TAP. The results showed that after the sham tDCS, participants acted more aggressively than before the stimulation, while after the active tDCS, alcohol abusers (but not the other groups) showed a decrease in aggression (pre–post comparison).

Lisoni et al. [191] tested the effectiveness of 15 sessions of bipolar bihemispheric tDCS over the DLPFC for reducing aggression in 30 borderline personality disorder (BPD) patients. BPD is characterized by a pattern of mood, behavioral, and self-image instability, including cognitive and motor impulsivity, reckless driving, suicidal behavior, and aggression [192,193]. Participants were randomly divided into two groups: real tDCS treatment (anode rDLPFC, cathode lDLPFC) and sham stimulation. Before and after the entire treatment, participants completed the BP-AQ. The results showed a reduction in both impulsiveness and aggression levels after the active but not sham stimulation.

A further approach was employed by two studies, which investigated the effect of bi-anodal tDCS over the DLPFC on aggression [194,195]. Choy et al. [194] randomly divided 81 healthy participants into two groups: one receiving monocephalic bi-anodal tDCS over the DLPFC with the cathodes placed at an extracephalic site (posterior base of the neck) and the second receiving sham stimulation in a single session approach. The assessment took part the day after the entire intervention: two hypothetical vignettes (written stories representing sexually or physically aggressive behavior) [196] were presented to the participants, who had to rate the likelihood that they would conduct the respective behavior and judge the immorality of those specific acts. The gender of the victim and offender were counterbalanced across the participant gender [196]. After the moral judgments, the VDT was performed. The results showed that active but not sham tDCS increased the perceived moral wrongfulness of sexual (but not physical) assault and decreased the perceived likelihood of committing both physical and sexual assault. Notably, the moral judgments partially mediated the reduction in the intention to commit sexual but not physical assault. Namely, participants who judged the sexual aggression vignettes as more immoral showed a higher reduction in the perceived likelihood of committing sexual assault. However, aggression as measured with the VDT did not differ between active and sham stimulation. The study conducted by Molero-Chamizo et al. [195] aimed to replicate the study of Choy et al. [194] in a sample of violent male murderers and non-murderer inmates. The crimes committed by non-murderers included different types of robbery with violence, drug trafficking fights, and gender violence. Participants were randomly divided into two groups: bicephalic bi-anodal tDCS over the DLPFC with the cathodes over the frontopolar cortex or sham stimulation, and received three intervention sessions. Participants completed the BP-AQ before and after the entire intervention, and a baseline measure of aggression was taken in a control group (non-prisoner sample). In the baseline measures, the physical aggression and hostility scores were higher in prisoners than non-prisoners, while an opposite pattern was found in verbal aggression (non-prisoners = murderers > non-murderers). The results of the intervention showed reduced anger, verbal aggression, and physical aggression scores after active but not sham tDCS in both prisoner groups and reduced hostility in murderers.

Wu et al. [197] tested the therapeutic effect of high-frequency rTMS applied over the lDLPFC on aggression and dementia symptoms with a sample composed of 54 patients with Alzheimer’s disease (AD) treated with risperidone. The participants were randomly divided into an intervention group (20 sessions of 20 Hz rTMS + risperidone) and a control group (sham rTMS+ risperidone). The coil position for lDLPFC stimulation was determined by moving the TMS coil 5 cm anterior to M1 (primary motor cortex, localized via motor-evoked potentials). Before and after the entire treatment, participants completed the Behavioral Pathology in Alzheimer’s Disease Rating Scale (BEHAVE-AD) [198], which comprises an aggression subscale. The results showed that baseline measures of aggression did not differ between the two groups. A statistically significant reduction in aggression in real intervention and control groups was observed after the treatment, showing an effect of risperidone or a non-specific effect of the stimulation treatment. Moreover, a specific effect of rTMS on aggression was observed since aggression significantly diminished after the intervention with real stimulation compared with the sham intervention group.

Only one study employed a TBS protocol for modulating aggressive behavior [199] in sixteen healthy volunteers. This study had a crossover design, and the interventions included inhibitory continuous theta-burst stimulation (cTBS) over the left DLPFC, cTBS over the right DLPFC, and sham stimulation. The coil position used to stimulate the DLPFC was identified via the strategy employed by Wu et al. [197]. Participants underwent two sessions with a one-week interval in between. In the first session, active and sham stimulations were performed with a 30 min interval in between and a randomly assigned order and laterality, and in the following session, participants were exposed to the opposite TMS and sham intervention. Following each intervention, participants completed the social orientation paradigm (SOP), which is a behavioral task that was developed to study individual differences in proactive and reactive aggressive behavior based on the point subtraction aggression paradigm (PSAP) [200]. The results showed an increase in both proactive and reactive aggressive behavior following cTBS over the lDLPFC compared with rDLPFC and sham stimulation conditions.

#### 3.1.3. MPFC

The first study by Gilam et al. [201] aimed to elicit and measure VMPFC activity enhancement during interpersonal anger via a single session of tDCS-fMRI and observe the modulation of aggression levels in 25 healthy volunteers. Participants were invited for two stimulation sessions (crossover design): anodal tDCS over the VMPFC with the cathode placed extracephalically over the right shoulder and sham stimulation in a counterbalanced order. During the stimulation, they were asked to perform an anger-infused ultimatum game (aiUG). The aiUG requires that a virtual proposer decides how to split a sum of money between himself and the participant, who decides whether to accept or reject the offer: thereby both players gain or lose the allocated money, respectively. Immediately before starting the game, participants received written insults from the virtual opponent, which aimed to provoke anger. After each stimulation session (active and sham within 6–9 days), participants completed the TAP. The results indicate that the active but not sham stimulation increased VMPFC blood oxygenation and fairness perception, as well as reduced self-reported anger associated with unfair but not medium or fair offers. Furthermore, aggression measures obtained using the TAP decreased over time during the active but not sham stimulation when the active stimulation preceded the sham stimulation but not when the active stimulation followed the sham condition. The order effect might be explained as follows: the effect of the stimulation might have been the prevention of provocation consequences in the first phase of learning and familiarization with the task. This is less likely when provocation had already produced effects in a previous (sham) session in which participants learned to behave aggressively. Finally, during the active stimulation, the anterior cingulate cortex (ACC) showed reduced activity in the case of unfair offers [201]. In accordance with other brain imaging studies [67,202], these results suggest a role of the MPFC in anger-driven aggression. More specifically, VMPFC and ACC showed significantly different activation levels during the active compared with the sham stimulation targeting the VMPFC. The direction of the effect of the stimulation over the VMPFC and ACC suggests that the first was associated with the suppression of unfairness perception and anger, whereas the latter was associated with the monitoring of prediction errors and reward delusions.

A study by Ling et al. [203] aimed to investigate the effect of a single session of HD-tDCS targeting the MPFC on aggressive behavior and antisocial intentions in a sample of 94 healthy volunteers. Participants were randomly assigned to active HD-tDCS over the MPFC (in the midline) or sham stimulation. HD-tDCS requires five small electrodes (skin contact area of approximately 2 cm^2^ each) in a 4 × 1 ring configuration with a central anode and surrounding cathodes. It is suggested that such an electrode configuration allows for higher spatial focality of stimulation [204,205]. Prior to stimulation, they completed a frustration task (12 unsolvable anagrams to solve), and immediately after the stimulation, they completed the VDT as a measure of aggression levels, as well as hypothetical vignettes [194] for aggression/antisocial intention assessment. The results showed no significant group differences in aggressive behavior measured using the VDT or antisocial intentions measured using hypothetical vignettes after the treatment.

The final study was conducted by Sergiou et al. [206], who aimed to examine the effects of 10 sessions of HD-tDCS over the VMPFC to increase empathy and reduce aggressive behavior in 50 forensic alcohol/cocaine-dependent offenders. Participants were randomly assigned to one out of two groups: HD-tDCS stimulation targeting the VMPFC or sham stimulation. Pre- and post-treatment assessments included the RPQ, the Rating Empathy Task [207], and the PSAP. The PSAP is a computer-based competitive game in which participants can steal points from an opponent, which is a behavior that indicates aggression [200]. Participants are provoked by the behavior of the opponent, who can also steal points from the participants. The results showed a reduction in the PSAP reactive aggression score following active but not sham tDCS. Additionally, both interventions (active and sham tDCS) showed a reduction in self-reported reactive (but not proactive) aggression and the total aggression score measured using the RPQ. No effects were found for empathy and other self-reported measures.

There are no studies available for TMS over the VMPFC.

### 3.2. Sample Size

A total of 942 participants took part in the included experiments. The studies were characterized by heterogeneous sample sizes ranging between 16 and 94 participants, M(sd) = 55.41 (+/−24.72). The only two studies involving less than 30 participants were also the only two that employed a crossover design [199,201]. Two of the three studies that did not find any statistically significant aggression modulation [185,203] had a higher sample size than the mean (respectively, 64 and 94).

### 3.3. Design

All included studies had a randomized sham-controlled single- or double-blinded design.

Only two studies employed crossover designs that compared active stimulation(s) to sham stimulation in different sessions for each participant, with a balanced order of conditions [199,201]. The first found a statistically significant aggression reduction, while the second one found a statistically significant aggression increase.

The remaining fifteen studies carried out a between-subjects design (comparing active and sham stimulation in different groups). Ten of them found a statistically significant aggression reduction [178,181,182,189,190,191,194,195,197,206], two of them found a statistically significant aggression increase [183,188], and three did not find any statistically significant effect [185,186,203].

### 3.4. Blinding

Of the eight single-blinded studies, five found a statistically significant aggression reduction [178,181,182,189,195] and two found a statistically significant aggression increase [183,199], whereas one did not find any statistically significant effect [185].

Of the nine double-blinded studies, six found a statistically significant aggression reduction [190,191,194,197,201,206] and one found a statistically significant aggression increase [188], whereas two did not find any statistically significant effect [186,203].

Blinding efficacy was explored in three double-blinded studies, of which two found adequate blinding of participants and researchers based on respective blinding indices [194,203] and one found adequate blinding of participants based on the guess of which kind of stimulation they received but did not explicitly test researcher blinding [188].

The remaining fourteen studies did not directly test blinding success with blinding indices.

### 3.5. Samples

Eleven studies involved healthy adult volunteers, where six of these describe a statistically significant aggression reduction [178,181,182,189,194,201], three studies found a statistically significant aggression increase [183,188,199], and two studies did not find any statistically significant effect [185,203].

Furthermore, the study involving forensic participants [195] found a statistically significant aggression reduction. Of the five studies involving clinical samples, four (including Alzheimer’s disease, BPD, alcohol abuse, and substance use disorder) found a statistically significant aggression reduction [190,191,197,206] and one study in military veterans with impulsive aggression problems did not find any statistically significant effect [186].

### 3.6. Stimulation Tools

Of the thirteen tDCS studies, nine found a statistically significant aggression reduction [178,181,182,189,190,191,194,195,201] and two found a statistically significant aggression increase [183,188], while two did not find any statistically significant effect [185,186]. Of the two HD-tDCS studies, one found a statistically significant aggression reduction [206], while the other did not find any statistically significant effect [203]. Both TMS studies found a statistically significant effect with, respectively, an increase and a reduction of aggression [197,199].

### 3.7. Stimulation Laterality

#### 3.7.1. Right-Hemispheric Enhancement or Left-Hemispheric Reduction in Excitability

Of the eight studies stimulating the right hemisphere with excitatory stimulation protocols, five found a statistically significant aggression reduction [178,181,182,189,190] and one found a statistically significant aggression increase [183], whereas two did not find any statistically significant result [185,186]. A study employed cTBS to reduce the excitability of the left hemisphere and found a statistically significant increase in aggression [199].

#### 3.7.2. Left-Hemispheric Enhancement or Right-Hemispheric Reduction in Excitability

Of the three studies in which the left hemisphere received excitatory stimulation, one found a statistically significant increase in aggression [183] and another found a statistically significant reduction in aggression [197]. A study employed cTBS for inhibition of the right hemisphere, which produced no effects [199].

#### 3.7.3. Bihemispheric Stimulation of Homologous Areas

Three studies employed bihemispheric stimulation of homologous areas (anode-left and cathode-right or vice versa). Of the two studies with a left-anodal and right-cathodal stimulation condition, one showed a statistically significant increase in aggression [188] and one did not find any significant effect [185]. Of the three studies that employed right-anodal and left-cathodal stimulation conditions, one found a significant reduction in aggression [191], while two did not result in significant effects [185,188].

#### 3.7.4. Medial or Bihemispheric Bianodal

Three studies employed anodal stimulation of medial sites. Two of them found a statistically significant reduction in aggression [201,206], while one did not [203]. Two additional studies delivered bihemispheric bi-anodal stimulation, with anodes over both the left and right hemispheres and cathodes over extracephalic or supraorbital areas, respectively [194,195]. Both reported a statistically significant reduction in aggression.

### 3.8. Intensity

In one of the thirteen tDCS studies, the stimulation intensity was set to 1 mA, resulting in a significant reduction in aggression [194]. Another study used 1.25 mA but did not find any statistically significant results [186]. Eight studies stimulated at 1.5 mA, of which five found a significant reduction in aggression [178,181,190,195,201], one found a significant increase in aggression [183], and one did not find any statistically significant effects [185]. Four studies stimulated with 2 mA. Three of these found a significant reduction in aggression [182,189,191] and one found a significant increase in aggression [188]. Both HD-tDCS studies used a stimulation intensity of 2 mA, with one finding a significant reduction in aggression [206] and the other not finding any statistically significant effects [203]. For rTMS and cTBS, the stimulation intensity was set to 80% and 100% of the motor threshold, respectively, and resulted in a significant increase and reduction in aggression, respectively [197,199].

### 3.9. Stimulation Duration

In the two TMS studies, the sessions lasted for 1 min and resulted in a statistically significant increase and reduction in aggression, respectively [197,199]. Two tDCS interventions lasted for 12.5 min and resulted in a significant reduction in aggression [182,189]. Among the two interventions that lasted for 15 min, one found a significant increase and the other found a significant reduction in aggression, respectively [188,195]. In the eight interventions that lasted for 20 min, five found a significant reduction in aggression [178,181,190,191,194], one found a significant increase in aggression [183], and one did not find any statistically significant effects [186]. One session lasted for 21 min and 45 s and did not find any effect [185], and another lasted for 22 min, resulting in a significant reduction in aggression [201]. Both HD-tDCS studies used 20 min sessions, with one finding a significant reduction in aggression [206] but without any statistically significant effects [203].

### 3.10. Aggression Measures

Of the eleven studies that employed only aggression tasks, seven used the TAP. Five of them found a statistically significant aggression reduction [181,182,189,190,201] and one found a statistically significant aggression increase [188], while one did not find any statistically significant effect [185]. A study employed only the VDT and did not find statistically significant results [203]. Three studies employed different tasks (CRTT, HSP, or SOP), and two of these found statistically significant aggression increases [183,199], while one found a statistically significant aggression reduction [178].

In four studies, solely self-report questionnaires were used as measures of aggression. Three of these employed the BP-AQ and two found a statistically significant aggression reduction [191,195]. One study employed the STAXI-2, which did not reveal a statistically significant effect [186], and one employed the aggressiveness subscale of the BEHAVE-AD scale, finding a statistically significant aggression reduction [197].

Finally, the two studies that employed both questionnaires and tasks found a statistically significant aggression reduction, but one only in the PSAP but not in the RPQ [206], and one only in the hypothetical vignettes but not the VDT [194].

### 3.11. Gender Effects

Fourteen studies involved both male and female participants. Eight of them analyzed gender differences, of which two found statistically significant gender effects on the modulation of aggression [183,189], while six did not [181,182,186,189,194,203].

In Gallucci et al. [183], the active stimulation over either rVLPFC and lVLPFC reduced gender differences, with females being as aggressive as males after the intervention, whereas in the sham stimulation groups, the females were less aggressive than males. In Dambacher et al. [189], in contrast, the active stimulation reduced proactive aggression only in males, leading to a reduction in gender differences.

Three studies did not include female participants [190,195,206], and one included only two females, which did not permit statistical comparisons [199].

## 4. Discussion

This systematic review summarized single- and double-blind randomized sham-controlled studies that addressed the modulation of aggression via NIBS in humans. We analyzed the literature investigating the effectiveness of non-invasive brain stimulation for modulating aggressive behavior, aiming to establish causal relations between cortical areas and aggressive behavior. We also aimed to identify the factors that influence the efficacy of stimulation in healthy, forensic, and clinical samples.

In most studies on healthy participants, only single-session interventions were conducted, and computer-based tasks were used to measure aggression, except for Choy et al. [194], who also used a self-report measure. In contrast, studies involving patients and forensic participants typically involved multiple intervention sessions (except for Weidler et al. [190], who employed only one session) and used questionnaires as the primary method for measuring aggression, except for Sergiou et al. [206], who also used a task.

Although the literature on aggression modulation through NIBS techniques is still growing, the analyzed studies suggest that the stimulation target in tDCS (defined as the electrode position of the international EEG 10–20 system, with the first letter indicating the cortical region, even numbers indicating the left, and odd numbers the right hemisphere, the letter “z” indicating the midline, and larger numbers indicating more lateral locations) [208] is a critical factor for successful modulation of aggression. Specifically, stimulation targeting the VLPFC at F6 and F5, the DLPFC at F4 and F3, and the MPFC at Fpz resulted in consistent statistically significant effects in modulating aggressive behavior (13 out of 14 experimental protocols), while other sites (i.e., either anodal stimulation over F7 and F8; AFz; or the crossing line between F8, Cz, T4, and Fz) did not (four out of four experimental protocols).

For tDCS, a stimulation target over one of these critical sites of the left hemisphere with the anode resulted in an increase in aggression (two out of two), whereas right-hemispheric anodal tDCS resulted in a decrease in aggression (six out of eight), regardless of the position of the cathodal return electrode. Even bihemispheric bi-anodal stimulation over these critical sites resulted in reduced aggression (two out of two), as well as stimulation of medial sites (FPz; two out of two), again regardless of the position of the cathodal return electrode.

Interestingly, the two TMS studies delivered the opposite results, with reduced aggression after the enhancement of left-hemispheric excitability and increased aggression after a left-hemispheric excitability decrease. This result, despite the small number of studies, might suggest a different mechanism of action for the two techniques on aggression modulation.

The specific stimulated PFC region within a hemisphere does not seem to constitute a relevant factor for differential effects since either DLPFC, VLPFC, or VMPFC modulation rather homogeneously altered behavioral aggression levels. Hence, the results preclude definitely supporting the hypothesis of a different role of these stimulated regions on aggression. Notably, the literature is preliminary due to the limited number of studies; the heterogeneity of methodologies, especially with regard to clinical samples; and the absence of consensus protocols. Additionally, several results are limited to specific conditions or subgroups, for example, Dambacher et al. [189] reported an active stimulation effect only for proactive aggression (and not reactive aggression) and only in men (not in women), while Weidler et al. [190] reported an effect limited to alcohol abusers but not extending to healthy volunteers. Furthermore, Gallucci et al. [183] reported an effect of stimulation over the rVLPFC only in women (not in men), and Riva et al. [181] described an effect only in unprovoked but not provoked aggression.

Additionally, the specific aggression measures were associated with the outcomes, with the TAP (six out of nine), PSAP (one out of one), HSP (one out of one), BP-AQ (two out of two), and SOP (one out of two) showing sensitivity to stimulation effects, whereas the VDT (zero out of two), RPQ (zero out of one), and STAXI-2 (zero out of one) did not. Nevertheless, due to a lack of comparative studies, we cannot make definite conclusions about the sensitivity of these measures.

Some studies suggest that NIBS interventions may be promising also for treating aggression in clinical samples, including Alzheimer’s disease [197], borderline personality disorder [191], alcohol abuse [190], and comorbid alcohol and cocaine abuse [206]. However, the results of studies on veterans with impulsive aggression and PTSD were less encouraging [186].

### 4.1. Relationship to the Motivational Direction Model of Frontal Asymmetry

According to the MDMFA, avoidance and withdrawal are associated with right-frontal activity, while anger-related approach behavior is associated with left-frontal activity. This model suggests that anger and aggression problems can be caused by frontal activity asymmetry, particularly an overactivation of the left PFC and/or an underactivation of the right PFC [32,74].

Of the twelve studies that employed lateral stimulations (either anodal unihemispheric or bihemispheric tDCS, or lateral TMS protocols), the results of four provide unanimous support for the MDMFA [178,182,189,191], two provided supporting results only of specific measures or subgroups [181,190], and two provided both supporting and non-supporting or contradicting results (i.e., support only with regard to the left excitation–right inhibition stimulation and not vice versa) [183,188]. However, two studies found only non-supportive results [185,186] and the two TMS studies found MDMFA-contradicting results [197,199].

Three studies, in which stimulation of medial regions was conducted, did not allow for interpreting results in relation to the MDMFA [201,203,206], and two studies challenged the theory, providing a modulatory effect of stimulation without involving a frontal asymmetry but employing bihemispheric bi-anodal stimulations [194,195].

Based on the presented results, one might conclude that both DLPFC and VLPFC provide inconsistent support related to MDMFA. Nevertheless, separate analyses of the results for specific samples (healthy vs. clinical/forensic) and electrode sites (resulted as effective vs. non-effective in producing a statistically significant modulation) show that studies that stimulated effective sites (F3–F4–F6–F7) provide more consistent support for MDMFA (six out of eight) than studies that stimulated other sites (zero out of two). Non-effective stimulation sites might constitute intervening factors for testing the frontal asymmetry hypothesis; however, there is insufficient data available to allow for definite conclusions. It is important to highlight that tDCS and TMS results are contradictory for the direction of aggression modulation since the overall tDCS results support the MDMFA, while the TMS effects oppose it. Finally, two studies reduced aggression with bihemispheric bi-anodal DLPFC stimulation, adding further challenges to the asymmetry hypothesis and offering new explanations for the relationship between hemispheric balance and aggression. Multiple mechanisms for aggression control (some characterized by hemispheric competition and some by hemispheric cooperation) might work in parallel. Further research is needed to close the knowledge gap about the complex relationship between stimulation laterality and excitability-enhancing or -reducing interventions and the direction of the effect on aggression.

### 4.2. Gender Differences

Previous research shows that males tend to be more aggressive than females [209,210]. These assumptions are based on a century of gender–aggression relationship investigations, with many mechanistic explanations for animals and contrasting results in humans [19,211,212]. In many species, males have a larger body size than females, often developing body parts specialized for fighting with other male specimens (e.g., horns in ungulates), and evolutionist theories interpret that as a result of sexual selection [2,213]. Even though tDCS studies report a differential gender effect of tDCS on aggression in different domains [147,214,215], the overall examined research does not unanimously support the differential gender effect hypothesis since only two studies out of eight found a gender moderation on the effect of neuromodulation, with females being more sensitive to aggression increase and males to aggression reduction. Furthermore, five studies found a gender difference in baseline aggression, with males being more aggressive than females in four studies and the opposite pattern in the fifth study.

### 4.3. Implications for the Aggression Control Network

According to aggression theories, aggression is an innate tendency sustained by neural and endocrinal substrates; shaped by the environment and socially acquired norms; and triggered by unpleasant experiences, thoughts, and emotions [1,15,19,28,76,213,216]. Affective components of aggression include negative emotions, such as anger and fear. Cognitive components of aggression rely on structured scripts of associated memories about contexts, expectations, thoughts, goals, emotions, and acts [1,17,18].

Neural mechanisms deputed to aggression control rely on a wide fronto-limbic network in which PFC regions associated with executive functions play a key modulatory role [63,68,217]. Some authors suggested that the modulatory role of the PFC is not a simple inhibition of subcortical areas associated with aggression genesis (i.e., amygdala, hypothalamus, PAG), but that respective modulation is based on cost–benefit analyses for impulsive behavior prevention [218]. Morality could play a primary role in modulating decision-making associated with aggressive behavior, as it was associated with self-control and the inhibition of impulsive behavior [219,220]. Morality involves different PFC regions, including the DLPFC, VLPFC, MPFC, and OFC [220,221], and Gilam et al. [201] showed that enhanced subjective moral wrongfulness about specific aggressive acts (such as physical or sexual violence) produced using DLPFC bilateral bi-anodal tDCS can reduce intentions to commit these.

### 4.4. Promising Stimulation Protocols

Based on the examined literature, the most promising stimulation protocols for aggression reduction with tDCS are bicephalic stimulation over the rVLPFC, targeting F6 with the anode and a contralateral supraorbital area or Oz with the cathode. Moreover, bicephalic stimulation over the rDLPFC targeting F4 with the anode and the return electrode positioned over the contralateral supraorbital or a contralateral homologous region showed promising results.

Furthermore, medial frontal anodal stimulation with both traditional and HD-tDCS showed promising results with respect to aggression reduction when targeting FPz but not AFz.

Additionally, bi-anodal tDCS over the DLPFC (F3 and F4) with cathodes either over bilateral supraorbital or extracephalic areas resulted in aggression reduction.

For aggression increase with tDCS, bicephalic stimulation over the lDLPFC (F3) and lVLPFC (F5) had the clearest effects: the first with the cathode over the contralateral homolog area and the second with the cathode over the contralateral supraorbital area. Stimulation over different targets showed lower-to-null effectiveness (i.e., F7; F8; AFz; and the crossing line between F8, Cz, T4, and Fz).

Based on the current literature, we are unable to make clear assumptions about whether a specific stimulation intensity or stimulation duration is superior to others. The same holds for electrode size, though, conceptually, the cathode should have the same size as the anode if the purpose is to produce an interhemispheric balance alteration, whereas the return electrode should have a relatively larger size if the purpose is to deliver a more focused active stimulation and to diminish or avoid the effects generated by a return electrode.

For TMS, the field is underexplored. Only two studies tested the efficacy of rTMS and cTBS on aggression modulation. Twenty sessions of high-frequency (20 Hz) rTMS over the left (but not right) DLPFC at 80% MT for one minute in Alzheimer patients showed an aggression reduction effect, while one session of cTBS over the left DLPFC at 100% of active MT for one minute in healthy participants resulted in an aggression increase.

### 4.5. Limitations of the Examined Literature

Some limitations of the examined literature include the heterogeneity of stimulation protocols (including intensity and duration of stimulation, electrode size, and target area).

Of the fourteen tDCS studies, one set the stimulation intensity to 1 mA, one to 1.25 mA, eight to 1.5 mA, and four to 2 mA. Both HD-tDCS studies set the intensity to 2 mA. For rTMS and cTBS, the stimulation intensity was set to 80% of the resting and 100% of the active motor threshold, respectively. For the number of sessions, ten tDCS studies followed a single-session approach, one conducted three sessions, one conducted five sessions, and one conducted fifteen sessions. The HD-tDCS studies employed single-session and ten-session approaches. Of the TMS studies, one employed two sessions, and the other twenty. Stimulation duration was set to 1 min per session for the rTMS studies. Two tDCS interventions lasted for 12.5 min, two lasted for 15 min, seven lasted for 20 min, one lasted for 21 min and 45 s, and one lasted for 22 min. Both HD-tDCS studies lasted for 20 min. As observed, the analyzed tDCS studies suffered from a lack of systematic dose titration (i.e., testing of the effects of different current dosages within participants for maximum benefits without adverse effects).

The sizes of the tDCS electrodes (anode/cathode) were 25/35 cm^2^ in five studies, 25/50 cm^2^ in one study, 35/35 cm^2^ in seven studies, and 35/100 cm^2^ in one study. Of the two HD-tDCS studies, one employed 2 cm^2^, and the other 3.14(π)cm^2^ electrodes. The TMS studies employed a figure-eight coil: one with a diameter of 70 mm, while the other study did not specify the coil size.

Concerning the electrode montage and site, the HD-tDCS studies employed ring montages with a central anode and surrounding cathodes. One of these positioned the anode over FPz and five cathodes over AF3, AF4, F3, F4, and Fz, whereas the other positioned the anode over AFz and four cathodes over FpùP1, FP2, F1, and F2. Of the thirteen tDCS studies, six employed a bicephalic bihemispheric montage with the anode over a lateral PFC site and the cathode over the contralateral supraorbital area. One positioned the anode over the F5 site; two positioned the anode over F4; three positioned the anode over the F6 site; and one of them positioned the anode over the crossing point between F8, Cz, T4, and Fz. Three studies employed a bicephalic bihemispheric montage with the anode over a PFC site and the cathode over the contralateral homolog area, two stimulated with the anode over F3 and cathode over F4, and three positioned the anode over F4 and the cathode over F3. One study employed a bicephalic bi-anodal and bi-cathodal montage, with the anodes over F3 and F4 and the cathodes bilateral over the supraorbital areas. Another study employed a bicephalic montage with the anode over F6 and the cathode over Oz. One study employed a monocephalic montage with the anode over Fpz and the cathode over the right shoulder, and another study employed a monocephalic bihemispheric anodal montage with the anodes over F3 and F4, while the cathode was positioned on the posterior base of the neck.

The heterogeneity of stimulation protocols did not allow for determining definite conclusions about the best-suited protocols at present. Additionally, the heterogeneity of outcome measures (as shown in the results section) complicates systematic comparisons between studies.

The analyzed studies are further characterized by a prevalence of between-subject (eleven out of seventeen) over mixed (four out of seventeen) and crossover experimental designs (two out of seventeen). This may constitute a limitation since between-subject studies comparing the aggression of participants exposed to different conditions need to assume equal baseline scores. A further problem relies on the fact that baseline aggression was measured with tools (aggression questionnaires, such as BP-AQ and RPQ) that in eight out of eleven studies were different from the outcome measures (aggression tasks, such as TAP and VDT). Employing different measures of aggression for baseline-outcome comparisons might be problematic since these often show poor correlations, especially if comparing self-report questionnaires and task results [222].

A possible source of divergence between tasks and self-report questionnaires may be attributed to the susceptibility of the latter to biases caused by social desirability and lack of self-awareness [223,224]. For example, Dambacher et al. [189] reported a positive correlation between baseline proactive aggression as measured using RPQ and outcome proactive aggression as measured using a TAP only in men but not in women, and no correlations for reactive aggression and total aggression scores. Similar results were provided by Choy et al. [194], who showed a positive correlation between baseline RPQ and outcome intentions to commit aggression but not VDT aggression scores. To avoid confounding effects of baseline aggression, it would be better to conduct a pre- and post-intervention comparison within subjects, as is done in mixed [186,190,197,206] and crossover designs [199,201]. These types of approaches permit reducing individual variability by comparing differences in repeated measures within subjects [225,226,227]. However, crossover designs may not be optimal for long-lasting clinical trials, especially when long-lasting carry-over effects are present since they require high costs and might be limited by interferences [226,228].

Most of the analyzed studies (fourteen out of seventeen) did not evaluate the efficacy of blinding, thereby precluding any meaningful estimation of the potential influence of participant and researcher expectations on the outcomes.

Even though stimulation of different frontal areas (i.e., VLPFC, DLPFC, and VMPFC) modulates aggression, the specific mechanisms remain unclear. Given the huge number of cortico–cortical connections of the frontal cortex [40,41,42,43,44], a common network might be activated even when stimulating different regions. This is coherent with previous literature showing therapeutic effects of brain stimulation targeting different areas that are part of a larger network and null effects for regions that are not part of these networks for several diseases [229], as well as similar working memory enhancements due to the stimulation of two different nodes of the working memory network (i.e., frontal and parietal targets) [230].

A more general limitation that is valid for tDCS may concern the activation of different networks due to the relatively poor spatial resolution of traditional tDCS employing 25–35 cm^2^ sponges. This arrangement was demonstrated to produce widespread dispersion of current throughout significant portions of the cortex [204,231]. Hence, some of the reported effects might not be restricted to the modulation of the nominal target. Imaging data would be useful for testing this hypothesis to investigate how wide this dispersion spreads and to what extent the spread of current relates to different network activation.

On the other hand, the included HD-tDCS and TMS studies do not show similar effects for different stimulation sites: HD-tDCS studies stimulated over two different sites (Fpz and AFz) and produced different results, whereas both rTMS and cTBS studies stimulated the same sites, finding convergent results (left DLPFC enhancement–aggression reduction and left DLPFC reduction–aggression increase). This pattern might be due to the higher spatial focality of HD-tDCS and TMS, which could lead to sharper find-or-miss results than traditional tDCS, which is able to modulate wide regions [232].

A limitation of the clinical and forensic studies is the lack of long-term follow-up measures to learn about the effect stability. Some studies suggest that a single session may have a limited impact on behavior, while multiple sessions may be necessary for more sustained therapeutic effects [84], suggesting a need for long-term monitoring. Additionally, clinical sample differences may lead to inconsistent results and poor generalizability, particularly when clinical populations or violent prisoners are involved.

Finally, some of the included studies suffered from protocol issues, further limiting data interpretation. Perach-Barzilay et al. [199] performed active and sham cTBS stimulation with a randomized order with a 30 min interval, which is not considered a sufficient washout period to avoid the interference of after-effects [161,233]. Instead, Choy et al. [194] collected the outcome measured the day after the intervention, not allowing for testing the immediate intervention effect.

### 4.6. Conclusions and Future Directions

In this systematic review, we examined key findings that provide insight into the effectiveness of NIBS techniques on aggression. Overall, the examined literature supports the suggestion that NIBS applied over the PFC over specific sites (i.e., F3, F4, F5, F6, and FPz) and with specific protocols is a promising tool to modulate aggressive behavior. However, future developments in the field of NIBS and advances in neuroscience will have to continue to improve intervention protocols, leading to new experimental applications and therapeutic approaches.

The use of NIBS combined with imaging techniques might provide individualized targeting and the registration of neural responses during online stimulation [234,235]. Of the studies included in this review, only Gilam et al. [201] employed such an approach, combining fMRI with tDCS. They found increased VMPFC blood oxygenation and reduced self-reported anger associated with unfair offers as a result of anodal tDCS over the VMPFC, suggesting a causal relationship between VMPFC activity and anger. Additionally, they found decreasd ACC activity associated with unfair offers, suggesting a differential role of this area, which may be related to the monitoring of reward delusion associated with unfair offers. These findings permit researchers to advance in the disclosing of neurofunctional relationships.

Furthermore, the relevance of dynamic phenomena of brain activity, such as brain oscillations and cross-frequency synchronization for cognitive processes, has been not exploited via stimulation studies in aggression [236,237,238]. Classical tDCS and rTMS alter spontaneous cortical activity, but their main effect is not interference with brain oscillations in a frequency-specific manner. Frequency-dependent effects may be obtained by employing transcranial alternating current stimulation (tACS), rTMS, or a combined approach at spontaneous endogenous frequencies [239,240,241,242].

Double-blind studies are recommended to control for the expectation effects of both the participants and experimenters since expectations might affect results in many ways, especially in aggression research [243,244,245].

Furthermore, TAP and BP-AQ seem to be the most reliable and sensitive-to-stimulation aggression measures. However, multimodal outcome measures (both tests and questionnaires) may be the optimal choice for shedding light on the effects of NIBS on specific explicit and implicit measures, and the relationships between these measures. For example, Chen [182] found no correlations between test and questionnaire measures of aggression. This suggests that these may measure different processes or a single process that may be influenced to different extents by the awareness of the participants. Questionnaires are completed with a certain degree of awareness, and the results may be influenced by explicit reasoning. In addition, physiological markers of aggression (e.g., fluctuations in salivary testosterone levels) might be relevant, and relatively direct and reliable outcome measures for testing NIBS effects on aggression, including dissociations between overt and physiologically implicit responses [216,246,247]. In conclusion, several studies highlighted the potential of NIBS techniques in the field of cognitive and social neuroscience for drawing causal inferences about the relevance of specific regions for cognitive, affective, and social processes, and for establishing new treatment approaches by modulating neural networks and observable behavior [81,84,110,231,248]. Yet, the potential of such techniques has not been fully explored for numerous psychological processes (including aggression), raising the need for high-quality single- and double-blind randomized sham-controlled studies. Moreover, systematic reviews of the literature and meta-analyses summarizing current works are needed for the identification of efficient protocols based on cognitive neuroscience knowledge.

The heterogeneity of the analyzed literature makes it challenging to compare the results across studies and draw general conclusions. However, the variety of stimulation protocols used in these studies also provides valuable information for future research on aggression modulation through non-invasive brain stimulation.

### 4.7. Limitations of this Review

The exclusive use of articles available in the database Pubmed may have limited the identification of further studies about NIBS effects on aggression. Moreover, we did not assess potential biases in the study design, execution, and reporting by employing tools such as the Cochrane Risk of Bias Tool.

## Supplementary Materials

The following supporting information can be downloaded at: https://www.mdpi.com/article/10.3390/life13051220/s1, Table S1: Keyword combinations employed in the Pubmed research. Table S2: A total of 942 participants were exposed to stimulation in the studies included in this review.
