# Supplementary material for: Non-Invasive Brain Stimulation for the Modulation of Aggressive Behavior—A Systematic Review of Randomized Sham-Controlled Studies
Source: Life (Basel). 2023 May 20;13(5):1220. doi: 10.3390/life13051220 (PMC10223137; doi:10.3390/life13051220)
Supplement: Supplementary file 1 [file life-13-01220-s001.zip › life-2375546-supplementary.pdf]

Table S1. Keyword combinations employed in the Pubmed research.

**Database**	**KEYWORDS**	**RESULTS**
PubMed	tDCS AND aggressive behavior	31 results
PubMed	tDCS AND aggression	39 results
PubMed	tDCS AND frontal cortex AND aggression	19 results
PubMed	tDCS AND parietal cortex AND aggression	1 result
PubMed	tDCS AND occipital cortex AND aggression	no results
PubMed	tDCS AND temporal cortex AND aggression	2 results
PubMed	rTMS AND aggressive behavior	22 results
PubMed	rTMS AND aggression	48 results
PubMed	rTMS AND frontal cortex AND aggression	16 results
PubMed	rTMS AND parietal cortex AND aggression	3 results
PubMed	rTMS AND occipital cortex AND aggression	1 result
PubMed	rTMS AND temporal cortex AND aggression	2 results
PubMed	Theta burst stimulation AND aggression	5 results
PubMed	Non-invasive brain stimulation AND aggression	18 results

Description of the selection process.

Two independent researchers took part in the selection process. All the items from each keyword combination search have been exported to a CSV file. 13 CSV files (one per keyword combination, minus one which obtained 0 results) have been merged into a single Excel sheet, and duplicates were removed through the function “remove duplicates” based on the PMID code. Afterwards, title and abstract screening were conducted, leading to the selection of 19 items. After the full-text assessment, three more contributions were removed (2 not yet published preregistered reports and a study not reporting comparisons between active and sham stimulation), and a further item has been found by checking citations of included contributions.

Table S2: A total of 942 participants were exposed to stimulation in the studies included in this review. DLPFC=dorsolateral prefrontal cortex; VLPFC= ventrolateral prefrontal cortex; VMPFC=ventromedial prefrontal cortex; rTMS=repetitive transcranial magnetic stimulation; tDCS= transcranial direct current stimulation; cTBS= Continuous theta burst stimulation; MT=Motor Threshold; aMT=active Motor Threshold; BPD= Borderline personality disorder; STAS= State-Trait Anger Scale; TAP= Taylor Aggression Paradigm; BP-AQ= Buss-Perry Aggression Questionnaire; RPQ= Reactive-Proactive Aggression Questionnaire; PSAP=Point Subtraction Aggression Paradigm; CRTT=Competitive reaction-time task; SOC=Social Orientation Paradigm; BIS-11= Barratt Impulsivity Scale-11; TBI=Traumatic brain injuries; N/A=not applicable; PTSD=Post-Traumatic Stress Disorder; SUD=Substance Use Disorder; STAXI-2=State-Trait Anger Expression Inventory-2; BEHAVE-AD= Behavioral pathology in Alzheimer's disease rating scale; PANAS= Positive and Negative Affect Schedule.

**Authors**	**Participants**	**M**	**M_{age} ± SD/ Range $_{age}$**	**NIBS type**	**Stimulation site/polarity**	**Electrode size/ TMS coil**	**Freq./ Intens.**	**Sessions/ Duration**	**SHAM**	Baseline aggression measure	**Measure**	**Outcome and direction**	**Study type**
Chen, 2018 [182]	32/Healthy participants	16	20.40 ± 1.33	tDCS	rVLPFC (F6)/anode; Oz/cathode	35 cm²	2mA	12.5 min	20s fade-in-fade-out stimulation	RPQ	TAP	Reduced aggression induced by active stimulation over the rVLPFC in both TAP proactive and reactive aggression scores	Between-subjects, single-blind, randomized sham-controlled design
Choy et al., 2018 [194]	81/Healthy participants	36	20.21 ± 3.31/ ≥18 years	tDCS	bilateral DLPFC (F3 and F4)/anodes; extracephalic /cathodes	25 cm² anodes, 35 cm² cathode	1 mA	20 min	30s fade-in-fade-out stimulation	RPQ	Intentions to commit aggression ; VDT	Reduced aggression intentions after active stimulation over the bilateral DLPFC (vs.sham), but no difference in the VDT	Between-subjects, double-blind, randomized sham-controlled design, follow-up
Dambacher et al., 2015a [185]	64/Healthy participants	39	21.89 ± 3.26	tDCS	G1: rVLPFC (F8)/anode, lVLPFC (F7)/cathode; G2: vice versa	35 cm²	1.5 mA	21.75 min	20s fade-in-fade-out stimulation	RPQ	TAP	No effect of tDCS over the rVLPFC or lVLPFC	Between-subjects, single-blind, randomized sham-controlled design
Dambacher et al., 2015b [189]	32/Healthy participants	13	22.14 ± 2.00	tDCS	rDLPFC (Faf)/anode; left eyebrow/cathode	35 cm²	2mA	12.5 min	20s fade-in-fade-out stimulation	RPQ	TAP	Reduced proactive (but not reactive) aggression after active stimulation over the rDLPFC only in men (vs. sham, vs. women)	Between-subjects, single-blind, randomized sham-controlled design
Gallucci et al., 2020 [183]	90/Healthy participants	45	22.27 ± 2.46	tDCS	G1: lVLPFC (F5)/anode; G2: rVLPFC (F6)/anode; Both: contralateral	25 cm² anode, 35 cm² cathode	1.5 mA	20 min	10s fade-in-fade-out stimulation	BP-AQ	CRTT, number of difficult numerical sequences and Tangram	Increased aggression induced by active stimulation over the lVLPFC after frustration, no effect of active stimulation over the rVLPFC. Higher	Between-subjects, single-blind, randomized sham-controlled design

					supraorbital/cathode						puzzles assigned to the partner	aggression in active stimulation (but no sham) in women (vs men)	
Gilam et al., 2018 [201]	25/Healthy participants	10	26.16 ± 3.63/ 21-33 years	tDCS	VMPFC (Fpz)/anode, right shoulder/cathode	35 cm²	1.5 mA	22 min	30s fade-in-fade-out stimulation	TAP	TAP	Reduced aggression induced by active stimulation over the VMPFC only in active-sham order (not in the opposite order)	Crossover, double-blind, randomized sham-controlled design
Hortensius et al., 2012 [188]	80/Healthy participants	40	N/A	tDCS	G1: lDLPFC (F3)/anode, rDLPFC(F4)/ cathode; G2: vice versa	35 cm²	2 mA	15 min	5s fade-in-fade-out stimulation	PANAS	TAP	Increased anger-driven aggression after left-anodal-right-cathodal active stimulation (vs sham) but no effects of right-anodal-left-cathodal	Between-subjects, double-blind, randomized sham-controlled design
Ling et al., 2020 [203]	94/Healthy participants	47, 80 %	18-28 years	HD-tDCS	VMPFC (Afz)/anode, Fp1, Fp2, F1, and F2/cathodes	Circular 2cm²	2 mA	20 min	30s fade-in-fade-out stimulation	BP-AQ	VDT	No effect of HD-tDCS over the Afz	Between-subjects, double-blind, randomized sham-controlled design
Lisoni et al., 2020 [191]	30/clinical (BPD)	12	40.3 ± 12.8/ 18-61	tDCS	rDLPFC (F4)/anode; lDLPFC (F3)/cathode	35 cm²	2mA	15 sessions/20 min	20s fade-in-fade-out stimulation	BP-AQ	BP-AQ	Reduced overall aggression score after active tDCS (vs. sham)	Between-subjects, double-blind, randomized sham-controlled design
Molero-Chamizo et al., 2019 [195]	41/forensic (violent murderers vs non-murderers)	41	36.2 ± 12.3/ ≥19 years	tDCS	bilateral DLPFC (F3 and F4)/anodes; bilateral frontopolar cortex (Fp1 and Fp2)/cathodes	25 cm² anodes, 35 cm² cathodes	1.5 mA	3 sessions/15 min	10s fade-in-fade-out stimulation	BP-AQ	BP-AQ	Reduced aggression scores induced by active stimulation over bilateral DLPFC in murderers and non-murderers group (vs.sham)	Between-subjects, single-blind, randomized sham-controlled design
Perach-Barzilay et al., 2013 [199]	16/Healthy participants	14	28±4.67/23-38 years	cTBS	lDLPFC vs. rDLPFC in two different sessions	70 mm figure-eight coil	triple-pulse 50 Hz at a rate of 5Hz/100%aMT	1 session/1 min	Placebo coil	SOP	SOC	Increased aggression after inhibition of lDLPFC but not rDLPFC (vs. sham)	Crossover single-blind, randomized sham-controlled design

Riva et al., 2015 [178]	80/Healthy participants	21 %	23.06 ± 4.36	tDCS	rVLPFC (F6)/anode, contralateral supraorbital/cathode	25 cm² anode, 35 cm² cathode	1,5 mA	20 min	8s fade-in, 5s fade-out stimulation	STAXI	Hot-sauce paradigm	Reduced aggression in socially excluded but not in non-socially excluded participants after active stimulation (vs. sham)	Between-subjects, single-blind, randomized sham-controlled design
Riva et al., 2017 [181]	79/Healthy participants	40	21.73 ± 2.38	tDCS	rVLPFC (F6)/anode; contralateral supraorbital/cathode	25cm² anode, 50cm² cathode	1.5 mA	20 min	20s fade-in-fade-out stimulation	N/A	TAP	Reduced unprovoked-proactive but not provoked-reactive aggression after active stimulation in violent video game players	Between-subjects, single-blind, randomized sham-controlled design
Sergiou et al., 2022 [206]	50/clinical (diagnosed alcohol and/or cocaine SUD)	50	37.40 ± 9.19/ 22-62 years	HD-tDCS	bilateral VMPFC (Fpz)/anode, AF3, AF4, F3, F4 and Fz/cathodes	circular π cm²	2mA anode, -0.4mA each cathode	10 sessions/20 min	30s fade-in-fade-out stimulation	PSAP, RPQ	PSAP, RPQ	Reduced PSAP reactive aggression score in active tDCS (vs sham), reduction in RPQ scores in both active and sham	Mixed between-within-subjects, double-blind, RCT
Smits et al., 2021 [186]	96/clinical (Military with PTSD, anxiety, or impulsive aggression problems), of which 45 tested for aggression)	89	18-60	tDCS	rIFG-rVLPFC (in between F8, Cz, T4, and Fz)/anode, left supraorbital/cathode	35 cm²	1.25mA	5 sessions/20 min	16s fade-in fade-out stimulation	STAXI-2	STAXI-2	No effect of tDCS over rVLPFC	Mixed between-within-subjects, double-blind, RCT
Weidler et al., 2022 [190]	51/clinical (Alcohol abuse), healthy smokers and non-smokers	51	18-60	tDCS	rDLPFC (F4)/anode, left supraorbital/cathode	35 cm² anode, 100 cm² cathode	1.5mA	20 min	20s fade-in-fade-out stimulation	TAP	TAP	Reduced increase in aggression over time only in alcohol abusers who received active stimulation, but not healthy volunteers or sham conditions.	Mixed between-within-subjects, double-blind, RCT
Wu et al., 2015 [197]	52/clinical (Alzheimer)	21	71.6± 4.8 [60-80]	rTMS	lDLPFC	figure-eight coil	20Hz/80 %MT	20 sessions/1 min	Coil turned 180 degrees	Aggressiveness subscale of BEHAVE-AD scale	Aggressiveness subscale of BEHAVE-AD scale	Decreased aggression score after active rTMS over lDLPFC, but not after sham.	Mixed between-within-subjects, double-blind, RCT
